# Quality of Life Philosophy III. Towards a New Biology: Understanding the Biological Connection between Quality of Life, Disease, and Healing

**DOI:** 10.1100/tsw.2003.103

**Published:** 2003-12-01

**Authors:** Soren Ventegodt, Niels Jørgen Andersen, Joav Merrick

**Affiliations:** The quality of Life Research Center, Copenhagen, Denmark

**Keywords:** quality of life, QOL, philosophy, human development, holistic medicine, public health, Denmark

## Abstract

This paper addresses (in a philosophical way) the complex and enigmatic interface between matter, life, and consciousness in modern medical science. The problem today in understanding living matter is not at the molecular level, but at the macro level where all molecular activities in the individual cell are coordinated, and especially at a higher level, where the activities of all the organism’s cells are coordinated. Although we understand very much of the body’s chemistry, we have only just started to get the gist of the tremendous organization of living matter. We are just beginning to acknowledge the enormous flow of information that is needed to make everything function in a healthy organism, including consciousness, where every cell does exactly what it has to do to make the organs function.A concept that seems to be able to bridge the scientifically very different domains of matter, life, and consciousness seems to be “biological information”. If a cell is seen as a liquid crystal in which the cell’s molecules constantly connect in firm mutual relationships only to dissolve again and become fluid and free, whenever the cell needs it, the backbone of the cell seems to be the information that organizes the cell. For example, in cell motion a cell is able to crawl with the help of a skeleton of fibers that can be created guided by biological information, whenever the cell needs the solidity provided by the fibers. The moment it has finished crawling or intends to crawl in another direction, these fibers will dissolve again. The fibers are made of millions of molecules that connect in an arranged pattern, and they dissolve when these molecules again let go of each other. How the cell precisely regulates such processes is today a complete mystery. How cells cocreate consciousness is also an enigma. All we can do is describe the cell and the organisms arising from its cells as filled with energy and information as well as an unbeatable ability to organize itself way down to the molecular level, where apparently the cell is in control of almost every single molecule.Our understanding today of how the information is stored and how it flows through living matter is still very limited. The source of the qualities (the qualia) characterizing the human being as a whole — like joy, love, motivation, consciousness, free will, wisdom, intuitive competence — is still practically unknown and scientifically unexplained, more than 50 years after science has turned itself towards these fundamental problems. We believe that we need a radically new biology and medicine to give the scientific explanations of the structure, dynamics, and quality of life, and of its consciousness.

## INTRODUCTION

In this paper we will take a closer look at the biological organism that is the foundation of human life. We do not mean biology in any narrow, materialistic sense, like biochemistry. Although biochemistry is a useful science, its description of life is still very crude and mechanical. Life cannot be reduced to chemistry and physics, which many modern biologists nevertheless try to do. As will be shown, chemistry and physics explain so very little of biological reality.

The recipe for a human being lies hidden in the depths of life. Figuratively speaking, one can talk about the essence of existence, the person as a whole, heart or soul. Intuition is a facility we can cultivate and use. Of course, to explain such complex phenomena from the world of human experience in biological terms, we need a rich and open-minded biology. In the future, one of the most important research programs will be the creation of such a broader biological science, which does not reduce life and awareness to simple chemistry.

In this article we take a philosophical perspective. We shall try to penetrate the mysteries of life and try to understand the extraordinary forces that characterize a living organism.

## MATTER, LIFE, AND CONSCIOUSNESS

The thoughts presented here come in part from the summer institute “The Spontaneous Order of Life” held in 1996 at the Niels Bohr Institute in Copenhagen[1] and from a group that studies the complexity of biological systems. The study group, which also took the initiative to the summer institute, is called “Matter, life, and consciousness” and it addresses three very fundamental questions that turn up if you try to understand any living organism: What is matter? What is life? What is consciousness?

One might imagine that medical science has a good answer to these three questions, since, typically, a physician will manipulate the living body with chemicals and physics to change the way we feel about ourselves. But this is not the case at all.

When the physicists in the group are asked what matter is — for example, professor Holger Bech-Nielsen, by many considered the best brain just now at the Niels Bohr Institute — they give oblique replies like this: “When we look to quantum mechanics for an answer to such a question we will discover that all our preconceptions about matter are nullified. As a matter of fact I am beginning to doubt whether matter really exists at all”[1]. And so on.

When we asked the group’s theoretical biologists what life is, we get the typical reply: “Today, we do not understand what life is. However, we have some models… oh, well, these models do not recreate anything remotely like life as we know it but still, it’s a beginning.” Answers like that[2].

When members who are knowledgeable are asked about consciousness —like the musician Peter Bastian, who wrote a best-selling book on the experience of music[3] —a typical reply is: “You cannot put it into words. Language cannot capture consciousness.” And so on.

By and by, we realize that the sciences do not possess proper answers to these three quite fundamental questions. Science has a great problem with understanding its own basis. The study group was started because of the suspicion that we need the various sciences together in order to tackle their fundamental problems satisfactorily. Perhaps the various sciences hold answers to each other’s problems and our real difficulty will turn out to be the demarcations. Perhaps the sciences need to be transformed through an internal dialog that will enable them to meet and provide a comprehensive and coherent theory of the world.

## PROBLEMS IN UNDERSTANDING REALITY

Science has great problems understanding reality[4,5,6]. The scientific disciplines are somewhat arbitrarily defined and their subject matters do not cohere across disciplines. For instance, it is probably impossible to understand matter, of which everything is created, without a deeper understanding of consciousness and our own brains that are attempting to understand reality. And without a basic understanding of matter, you are probably unable to understand life, because the living is part of the creative principles that are found in matter itself.

Life was created on the basis of a driving, developing force in matter. Perhaps we are not even close to understanding this force. Biology is usually understood on the basis of chemistry and physics. Maybe we should turn this process upside down and start learning physics and chemistry from biology. Maybe life itself is best able to disclose the profound possibilities and properties of matter.

Quite possibly, consciousness and the brain cannot be understood unless we have a different and more fundamental insight into life than the one science possesses today. There is much talk about all these models of artificial intelligence, neural networks, or adaptive programs, but they totally lack the basic creative dynamic we know as awareness or the mysterious light found in living organisms, which is the basis of awareness itself.

So, as far as the phenomena of life are concerned, we draw a blank with the models we use in biology today. We need new principles if we want to explain the very strange phenomena that characterize anything living, such as the subjective experience of one’s life and its expression on quality of life or in disturbed processes in the organism. But first, let us talk a little about the understanding of life held by most of today’s biological scientists.

## DNA ONLY EXPLAINS VERY LITTLE OF LIFE

Traditionally, life is explained from biochemistry: It is based on DNA. In the center of every single cell is a very long, thin structure that contains our hereditary material, our genes. Directly and indirectly, this DNA molecule contains the recipes for all the other molecules in the body. When the DNA is “run” or activated, all these other molecules are created from the DNA’s recipes. Scientists know quite a lot about this today.

However, science does not know much about what governs the activation of the DNA. Also, little is known about how the molecules whose creation the DNA controls are directed to the right places in either the cell or the organism. The DNA tells you what substances there are in the cell or organism, but not *where* they are or *what* they do, that is, how the cell or the organism is structured and what processes are taking place and when. So the DNA molecule does not explain the shape of the body or the function of the organs. Neither does it explain complex, high-level processes like consciousness or communication between living organisms. How cells communicate in order to know their individual place in the scheme of things is a major, unanswered question.

Within the science of biology there are various trends, schools of thought, or systems of faith, if you like. The most popular system maintains that the living state can be reduced to chemistry without any problems. According to this credo, you will think that you understand a flower if you know all the molecules within it and know how these molecules influence each other, just like you understand how a car works by taking it apart, bit by bit.

According to this theory of life, everything living is based on what is called self-organizing processes, in which the molecules themselves can create and build the finished organism. To imagine that the flower is pure chemistry is like imagining that the car can assemble itself, because the bits and pieces are "clever" enough to do this by themselves with a little help (like a good shake-up). But this is not enough, the finished car must also be able to drive itself up and down a busy street.

Even though it cannot be ruled out that in the final analysis, life can be explained as "molecular self-organization", it is still our opinion that life needs much more information than what is found in molecules. The point is that information is needed to put the car together. This information is only found in the brains of the manufacturer and the auto-mechanic. Likewise, information that guides the organization of life's molecules must somehow be present in every living organism.

In our opinion, this information is associated with anything living in such a complex way that completely new laws of nature must be discovered, before we can give a reasonably adequate account of the living state. Maybe some form of complicated quantum chemistry or a whole new concept of the physical field is needed. Apparently, the information is stored in an unknown and very peculiar, dynamic way that allows it to be marshaled from the organism to run and guide the living processes at all levels, from molecule to organ and organism. This information can even guide the organization of whole societies of organisms, just look at the ants. The central point here is that the biological information can also guide human beings, although it probably happens in a far more dynamic way, that is, through human consciousness.

Researchers who reduce all living organisms to chemistry only focus on certain chemical aspects of these organisms. However practical it is to know these chemical aspects, it seems rather trivial if compared with the living organism's highly advanced processing of the biological information.

Our understanding of life is based on the concept of biological information. This concept may help us clarify complex phenomena like the living, highly developed organism's ability to be conscious. Consciousness is an example of emergence. Completely new properties emerge in a whole when its elements interact in complex ways[7]. A very simple example of emergence in a physical system is the transition from individual gas molecules to the properties that characterize air, such as pressure and temperature. From everyday life, we know about cars in the streets, but traffic jams are patterns that emerge from the interactions of many individual cars.

Living organisms are very much characterized by emergence. Life is built up through a large number of levels, with the still higher levels possessing ever more complex traits, including the qualities known so well in the human world: love, hate, consciousness, the subconscious, reason, and intuition.

## DNA AND ILLNESSES

Thus, it seems naive to believe that DNA and biochemistry explain the riddle of life and all the peculiar qualities that are tied up with living organisms. Yet this belief is very widely held. Billions of dollars have been expended by an international research project to decipher the entire three billion "letters" that constitute the molecular recipes in the human DNA.

We already know that certain diseases are caused by "defects" in some of these recipes — bad genes — which means that important molecules in the body are either missing or not working properly. Take an illness like oligophrenia phenylpyrouvica in which a certain enzyme is missing that normally biodegrades the amino acid phenylanine in food. The result is a build-up of a dangerous substance, PKU (phenylketonuria), in the blood that can damage the brain. Thus, this genetic defect can render people carrying the gene intellectually disabled unless they avoid the amino acid for the rest of their lives. If you screen every newborn baby's urine for PKU, the disease can be prevented. Unfortunately, however, a simple correlation that can explain a disease and lead to its prevention is the exception rather than the rule. Most diseases have proved to be much more complicated.

Instead of rejecting the idea that genes are the cause of most diseases, scientists try to save their basic understanding with the hypothesis that a *combination* of bad genes is the cause of illness. With a massive mapping of the DNA that systematically reveals all the defects in the molecular recipes, scientists and a great number of medical doctors hope we will eventually understand all the major human diseases such as schizophrenia, Alzheimer's, senile dementia, breast cancer, arteriosclerosis, and obesity.

We suspect that medical research is on a wild goose chase and that billions of research dollars could have been put to better use. For instance, it is often pointed out that molecular cancer research has yielded few results in the battle against cancer over the past 30–40 years. The problem stems from trying to reduce life to a question of chemistry and nothing else.

## WHAT IS LIFE[8]?

To understand what life is let us start with studying tiny, simple creatures. Take a small jellyfish, a freshwater polyp called Hydra. It consists of three layers of cells and is probably akin to the first creatures on earth[9]. Hydra can swim, catch brine flies with its tentacles, turn small somersaults, and many other things. It can also learn. But it does not have a brain, just a simple network of nerve cells that connects an outer layer of cells, which acts as both skin and muscle. Inside, Hydra has a layer of cells that cover a primitive stomach, which secretes digestive fluids and absorbs nutrients.

This animal is extremely simple, but it can do a lot. Hydra reproduces by budding, just like many plants extend cuttings. If you cut Hydra into small pieces each of these will turn into a new, small polyp, a miniature copy of the first one. When a lump of cells forms a Hydra, all the cells talk to each other about what a Hydra looks like, they study the recipe for a Hydra that lies hidden in the cells, and then they agree on who is to do what, where, and when. As soon as the cells have established the correct order, the new Hydra will function and it can swim around and catch flies. All the knowledge the creature needs is contained in the biological information, ready to use.

Hydra is a multicellular creature evolved from single-celled organisms, which learned to communicate with each other so well that they were able to establish well-functioning colonies. Hydra is made up of cells, each of which is a small animal. Various organisms exist between single- and multicellular organisms, for instance, a slime fungus that can also be found as parasitic amoebae or multicellular "snails". The multicellular form occurs when the amoebae need to get away in a hurry, because there is no food left where they live. They are lost on their own, but together they can move far and fast.

If the "snail" does not find anything to eat, it will form a new structure and become a small fungus with foot, stalk, and sporangium. Some of the cells become small, hard, light spurs that can be spread for miles by the wind and thus survive to found a new colony of cells. Most of the cells in the colony are sacrificed in order that a few may survive.

These examples illustrate the way we understand life: Life is profoundly intelligent, extremely dynamic, and totally geared towards serving the needs of the whole. Compared to far more complicated living creatures like man, these small primitive cell colonies are nothing. Man consists of approx. 10,000 billion cells, but today these cells can still function as small independent beings. Several experiments will show that the cells in the body really are small animals. Take a very tiny bit of the index finger and put it in warm sugar water. After a few days, you will see in the microscope that many small creatures are crawling around at the bottom of the glass. This is not because the sugar water had dirt in it. Many of the finger's cells (such as fibroblasts) have actually left the community in the finger and are roaming free at the bottom of the glass where they apparently seem to thrive, eating, budding, and crawling around. It appears to me that the reason they are able to survive on their own is because they remember life as independent single-celled amoebae at the bottom of the primeval sea billions of years ago.

The body can heal its wounds much the same way the Hydra recreated its order. A cut finger often heals perfectly, because the cells can study the recipe that was used to shape the human being to begin with. The healing may occur in the same way that the original form developed, but still it will often be a perfect healing.

However, large organisms such as humans often encounter problems due to size. The forces that organize living matter find it difficult to recreate structures so enormous (as seen from a cell's perspective) as an arm, for example. If we lose an arm or a leg they will not grow out again whereas small, more primitive animals with legs like newts or cockroaches grow new legs without problems.

The recipe for a human being maintains the body's shape all through life. The cells in the body keep dying and being replaced. Many cells only live for a few weeks. But still we exist for years, because we are not the cells, but the order created by the colony as a whole. Through life we can grow and hold more cells or we can shrink and consist of fewer cells — either way it is irrelevant to personal identity. We are who we are with more or fewer cells as long as order is maintained and the recipe for being human is unfolded.

## THE FLOW OF INFORMATION IN LIVING MATTER

Characteristic of living matter is the incredible ability to store and reclaim information and communicate this information to other living beings. If we look at the formation of the human fetus during pregnancy, it is clear that today, scientifically speaking, we hardly understand anything of this miracle.

If we study a random cell in the organism, we will find that it contains enormously complicated structures and shapes that are still quite inexplicable to science.

You can imagine the cells as a liquid crystal[1] in which the cell's molecules constantly connect in firm mutual relationships only to dissolve again and become fluid and free whenever the cell needs it. For example, a cell is able to crawl with the help of a skeleton of fibers that can be created whenever the cell needs the solidity provided by the fibers. The moment it has finished crawling or is crawling in another direction, these fibers will dissolve again. The fibers are made of millions of molecules that connect in an arranged pattern, and they dissolve when these molecules again let go of each other. How the cell precisely regulates such processes is today a complete mystery. All we can do is describe the cell as filled with energy and information as well as an unbeatable ability to organize itself way down to the molecular level, where apparently the cell is in control of almost every single molecule.

The problem today in understanding living matter is not at the molecular level, but at the macro level where all molecular activities in the individual cell are coordinated, and especially at a higher level where the activities of all the organism's cells are coordinated. Although we understand very much of the body's chemistry, we have only just started to get the gist of the tremendous organization of living matter. We are just beginning to acknowledge the enormous flow of information that is needed to make everything function in a healthy organism, where every cell does exactly what it has to do to make the organs function.

The organism contains the whole history of its evolution. When a fertilized egg (the first cell), becomes a human being, the evolution of species to the human being over the past billion years is repeated in rough form over a few months. From its single-celled existence, the fertilized egg starts a colony of cells that soon features the polyp's three layers. At progressively later developmental stages, the fetus resembles in turn a fish, an amphibian, a mammal, a monkey… and finally a human being emerges. Our understanding today of how the information is stored and how it flows through living matter is very limited.

## THE LEVELS OF LIFE

It is characteristic of life that it is organized at several levels. The cells are made up of a number of very tiny organs, which supposedly were independent organisms to begin with and later were fused into the first large cell. The cells join up and form the organs that make up the organism, which again, together with other organisms, form communities.

Humans have bones, muscles, connective tissue (motor functions), and internal organs: a heart that keeps the blood circulating, lungs that exchange carbon monoxide for oxygen, and kidneys that filtrate the blood. They also have a stomach and a digestive system that absorb food, glands that regulate the body's metabolism, and sexual organs that handle reproduction. Furthermore, we have a nervous system with senses that connects the brain to the world, and a peripheral nervous system that connects the brain to the body.

Every level possesses emergent properties that do not exist at previous lower levels. The cells have qualities that do not exist in the molecules and the organs can do things that the cells cannot do on their own. Together the organs make up the organism with powers that enable the organism to enter into relations with the external world.

In the same way, the outside world is organized through a number of levels. A person has relationships, family and friends, relations at work, in the local community, society at large, and finally the global ecosystem. As we see it, the forces that connect cells and organs to an organism in principle are the same as those that connect the organisms in a larger community.

Although life at the micro-level usually consists of cells, there are exceptions. Our blood platelets that help blood coagulate are alive, but they are not cells because they originate from the ligation of bits from one enormous cell that produces the blood platelets. A muscle fiber holds numerous cell nuclei and is therefore not a cell, but a complete and real fusion of many cells.

You could say that living matter is better described by the flows of information that connect all levels of life than by the cell itself — that life's magic is all about the relationships and exchanges of information that connect living matter with the entities at all levels of reality.

## FROM INFORMATION TO LOVE

One way of describing the human organism is that it is a highly organized blob of mucous made up of billions and billions of small creatures that talk to each other. These cells communicate so efficiently that they are able to dissolve the distance between each other and fuse into the entity we know as a person (me), an organism with consciousness.

Parallel to this process at the micro-level, we know about communication at the macro-level between people, which reduces distance. The picture is complicated by the fact that people can communicate in several ways, through words and reason (which often increases the distance between people!) and through feelings. When we speak with each other we attune our view of the world and our sense of reality, because this is essential for cooperation and the practical organization of the world. But when we just talk to each other — when we exist in the same space — our organisms exchange enormous amounts of information that is never expressed in words, but which makes us really close and able to know each other properly.

To us, love is a direct expression of the process by which living matter reduces distance through communication. You may feel so close to a loved one that you experience a kind of unity, where you understand each other completely. However rarely this occurs, we have all caught a glimpse of it. Such love is a potential in all of us, and we all long for it and hope to experience it in life.

From a scientific perspective, living matter's ability to communicate is a very peculiar phenomenon. All the cells in the colony are in direct communication with each other and this communication organizes a community, which corresponds to the information stored through the history of evolution as the recipe for the organism. Through this biological information, every organism appears to be in contact with all the other organisms, both near and far. When you experience that you are one with your partner, it is because of life's enormous ability to exchange information. We think that when we communicate intensely or, actually, when we are just together in the same room and communicate, our bodies and our subconscious will know all about each other. Unfortunately, our reason is far too coarse and slow to keep up. The exchanged information is far too voluminous and complex. Nonetheless, this means that our life just below the surface of consciousness is filled with intimacy and information flows.

Such a view of life renders much mysticism redundant. Phenomena like synchronicity (you think of a person you have not talked to for years and the phone rings — and who is it?)[10], mind-reading (you know what the person is thinking before he says it), extrasensory perception (ESP), and clairvoyance are things that a lot of people experience and wonder about. They are probably an expression of life's ability to communicate without words. Life really is mysterious, but there is no reason to make it more mysterious than it already is. All life communicates and, actually, this is what living matter does best of all. And only an infinitesimal small part of communication happens through words and reason.

## FROM BIOLOGY TO SOUL, HEART, AND INTUITION

We have used words like heart, soul, the core of existence, the depth of life, and whatever else that fit the context. This abstract center has endowed human beings with qualities like love, intuition, wisdom, will to live, and vital energy. We have described the ability to live from this center of your life's circle with expressions like being centered, in balance, knowing yourself, resting in yourself, knowing the meaning of life, and having established a bridge between the innermost soul and the world.

However meaningful these terms have seemed, the use of such words is not sharp or precise enough to scientifically explain the correlation between quality of life and illness. Therefore, it is time to clarify the nature of this center in a more exact way. We do not see the soul as a little spirit that is temporarily housed in the body (like the genie in the bottle), but as something that our reason finds extremely abstract and hard to grasp. When we apply the biological view of human beings, which is our favorite view, they appear as a simple, beautiful, and ultimately understandable being. To a great extent it is possible to translate the psychological and spiritual phenomena into a biological frame of understanding without violating these phenomena.

The center of the soul, heart, or existence corresponds to the dialog that proceeds at all times between all the cells in the organism. Intuition corresponds to the flows of information in living matter that can be sensed wordlessly so that they may guide our acts in life. Love is an expression of a fundamentally social quality in living matter, which is caused by a natural tendency to exchange information. Love between the sexes happens when this social quality is combined with a long evolutionary tradition for procreation of the species.

The will to live can be seen as all the cells' joint wish to live. Vital energy is the driving force in the cellular colony that tries to manifest the recipe for man in the world. To love oneself expresses the fact that there is no distance between our minds and our health, i.e., that our view of life does not deny or conflict with the life that exists deep down in us. To find the meaning of life means to be perfectly present in the world with sound and close relations to reality at all levels. Therefore, to find the meaning of life is like building a bridge between the innermost existence and the surrounding world. When we really discover ourselves, we discover coherence. Because coherence, communication, and transfer of information are what life is best at.

All living matter communicates and creates an extremely comprehensive community, much like a whole organism, on earth. Therefore, you can be joined to the world in a subtle way so that you love the earth with all your heart and become one with the world around you. If it really is true that all living matter continuously exchanges information, then, in principle, all knowledge about the world is at our disposal here and now, if we are only sensitive enough. In other words, one may speculate that our intuition in fact mines this unlimited source of knowledge about the world.

## QUALITY OF LIFE AND THE BIOLOGICAL ORDER

Now let us return to the question about what it means to experience quality of life. How are we able to discover how we feel deep within our souls? How is it possible for us to gauge our innermost selves? To find out how happy we are, how our inner balance is doing, and how much meaning there really is in our life?

You can do a superficial stocktaking just by reckoning: If life is what you want it to be, you are happy and content. But how do you really take stock of these subtle, comprehensive conditions like happiness and personal meaning that involve every fiber of your existence, every tiny cell in your body? Our answer is that when we sense our innermost selves, what we gauge is the state of our organism, which is the same as the state of the dialog between all the cells. You could say we try to estimate how well our recipe for being a person has been realized.

If the organism (and the levels of life outside and inside) corresponds with life's profound recipe for a human being, life seems to overflow with meaning and everything makes sense or there is an overall consistency. If the recipe for a human being is poorly carried out we are disconnected, weak and vulnerable inside, and life is neither happy nor meaningful. One's quality of life is low in the deep, existential dimensions.

Our souls are inside this order, this subtle dialog between all living matter that exists inside us, all our cells. When we feel inside ourselves, we take stock of the state of our soul, the order of our organism, and the harmony of our inner biological communication system. A sound state of affairs is experienced as meaning, coherence, happiness, and joy, while conflicts and disharmony in the subtle order of the organism feels like just the opposite.

It is important to note that we can easily fool ourselves into believing that we are fine without even when we are not. We can lie to ourselves to make us content with even a miserable life. What we are talking about here is the feeling deep inside of you, not the one on the surface.

## QUALITY OF LIFE AND ILLNESS

It is now clear that illness can be seen as a breakdown in the subtle order of the organism. Illness happens when the cells do not know exactly what to do and the reason that they do not know is that there is disharmony in the dialog between the cells. In this connection, it is obvious to think about how we picture reality, life, and ourselves. If this picture is in contrast with the recipe for a human being, which is contained deep within our biology, there is an inner conflict that we have already encountered as our poor relationship to ourselves. You do not love yourself and life.

Many people find it surprising that there appears to be a connection between consciousness and the organism's biological order. Several scientific experiments[11,12,13], however, have shown that there is such a connection. If, for instance, you can make hypnotized people change their opinion about, say a spot on the skin, you will often see that the skin does change[12]. If the person believes he gets burned, a blister will appear even though the temperature was unchanged. The opposite can also be true, you can suppress a blister in spite of strong heat if you make it very clear to the person that nothing is wrong. If a person believes that he has been injected with tuberculin, which creates a strong immunological reaction, this reaction will happen even though only salt water has been injected[12].

It is obvious that people who walk on glowing embers use this phenomenon in order not to get burned despite strong, local heat. We have seen this in various places in the world. There is a puzzling proximity between consciousness and the biological order that surprises those who see the human being as pure chemistry. Because how is it possible for the consciousness, located as it is in the brain, to prevent blisters on the toes? However, if we see the organism as a whole where everything is connected through the cells' communication with each other and if we imagine consciousness as something that results from this dialog between the cells, everything becomes a little less puzzling.

This proximity between consciousness and the biological order can be found in many different experiments. Take the famous study where a group of cancer patients met in small groups to talk about their lives and problems. These patients survived twice as long as those in a control group that did not meet. Members were assigned to each group randomly, and they were all equally sick when they started[14]. It also turns out that people who suffer from thrombosis can postpone the time of the next thrombosis if they engage in attempts at personal growth[15].

Many of the illnesses spread across the modern western world are of a type where the flesh just about falls from the bones without any apparent external or internal (genetic) reason. You just need to think of arthritis, multiple sclerosis, breast cancer, and colitis to get the picture. In most of the chronic diseases, there is no known etiology — no known reason — like a known provocative factor in the environment or a genetic weakness known to cause the disease. One may speculate that the main cause of these illnesses is a lifestyle that basically disrupts our own organism and ends up causing our ruin and death, and all because we have squandered the subtle order of our lives.

Perhaps people that become ill actually are the ones who, through life, did not take care of their experience of happiness and a meaningful life? And those who become well again could be the ones who have managed to reclaim the meaning of life[16]? This explanation suggests that you may understand illness by imagining the cells' communication and the biological information. Maybe we need not to think about physics or chemistry at all in order to understand what happens when we become ill and what is needed to become well again. Maybe we just need to look at the correlation between quality of life and the biological order?

If this is correct, there is every reason to revise your life if you should become seriously ill. Perhaps an illness is your real chance to figure yourself out, discover life's coherence, and revise ill-suited views and preconceptions[17,18,19]. As long as the recipe for the human being is intact and active in our innermost selves it is possible, in principle, to become well again.

## OUR LOVE FOR LIFE

Life is full of battles and competition and worthy opponents. Many have made this the most important aspect of life and interpreted life as a continuous battle for survival. However, to us, our love of life is the primary quality. Competing with other living creatures in the battle for survival and realization of life is secondary.

From life's point of view, animals and plants are connected to each other and the surrounding world in a direct and uncomplicated way. But for every person, it is a mission in life to discover exactly this uncomplicated and direct way of being part of the world. Personally, we find that this proximity is like a deep love for life and the world around us. To us, the force of this connectedness in life is the raw material of which human love is made.

The love of life is one of the most important sources for solving our problems. If my best friend should die, I shall find a new one when I am through grieving over his loss. If my wife should leave me or I leave her, I shall still have my love of life. But without the love of life itself, the gigantic whole of which we are a part, there will be nothing much to live for. With that love as the backbone of our existence we can still be toppled, but never really knocked down and never really beaten by anything, but death itself.

Our individual worlds may tumble down and there will still be hope if the backbone in life is love of life itself. When you discover life within yourself — in your innermost self — you do not find your ego or an isolated island. On the contrary, you find a unity, a coherence, and life's own, eternally growing forces[20]. The greatest life we can live is a life in the service of these forces far greater than us.

## CONCLUSION

The human organism is a highly organized blob of mucous made up of 10,000 billions of small creatures, the cells, that communicate so efficiently that they are able to dissolve the distance between each other and fuse into the entity we know as a person (me), an organism with consciousness.

Parallel to this process at the micro-level, we know about communication at the macro-level between people, which reduces distance. The picture is complicated by the fact that people can communicate in several ways, through words and reason (which often increases the distance between people!) and through feelings. When we speak with each other we attune our view of the world and our sense of reality, because this is essential for cooperation and the practical organization of the world. But when we just talk to each other — when we exist in the same space — our organisms exchange enormous amounts of information that is never expressed in words, but which makes us really close and able to know each other properly.

To us, love is a direct expression of the process by which living matter reduces distance through communication. You may feel so close to a loved one that you experience a kind of unity, where you understand each other completely. However rarely this occurs, we have all caught a glimpse of it. Such love is a potential in all of us, and we all long for it and hope to experience it in life.

From a scientific perspective, living matter's ability to communicate is a very peculiar phenomenon. All the cells in the colony are in direct communication with each other and this communication organizes a community, which corresponds to the information stored through the history of evolution as the recipe for the organism. Through this biological information, every organism appears to be in contact with all the other organisms, both near and far.

We need a radical new biological and medical science to explain biological information to such an extent that quality of life, love and consciousness, qualia and joy will be understood. Such a science will give us the bridge between consciousness and biological order at the cellular level, we need to explain the mysterious strong connection between quality of life and health and disease[21,22,23]. We suspect such a new, more holistic medical science to explain the hundred or so scientific studies indicating that love and intimacy often is the best medicine[24].

## ACKNOWLEDGMENTS

This study was supported by grants from The 1991 Pharmacy Foundation, as well as by supplementary grants from Goodwill-fonden, the JL-Foundation, E. Danielsen and Wife's Foundation, Emmerick Meyer's Trust, the Frimodt-Heineken Foundation, the Hede Nielsen Family Foundation, Petrus Andersens Fond, Wholesaler C.P. Frederiksens Study Trust, Else & Mogens Wedell-Wedellsborg's Foundation and IMK Almene Fond. We gratefully acknowledge the critical scrutiny and expert linguistic assistance of Ib Ravn, Ph.D. and also the assistance of the philosopher Maximilian Kroman. The research was approved by the Copenhagen Scientific Ethical Committee under number (KF)V.100.2123/91.

## BIOSKETCHES

**Søren Ventegodt, MD,** is the Director of the Quality of Life Research Center in Copenhagen, Denmark. He is also responsible for a Research Clinic for Holistic Medicine in Copenhagen and is a popular speaker throughout Scandinavia. He has published numerous scientific or popular articles and a number of books on holistic medicine, quality of life, and quality of working life. His most important scientific contributions are the comprehensive SEQOL questionnaire, the very short QoL5 questionnaire, the integrated QOL theory, the holistic process theory, the life mission theory, and the Danish Quality of Life Research Survey, 1991–94 in cooperation with the University Hospital of Copenhagen and the late pediatric professor Bengt Zachau-Christiansen. E-mail: ventegodt@livskvalitet.org. Website: www.livskvalitet.org/

**Niels Jørgen Andersen, MSc,** Professor, Department of Innovation and Economic Organization, Norwegian School of Management. This department conducts research and provides teaching in central topics related to innovation, business development, management of global companies, business history, and economic organization. Research activities within the Department are related to four core subjects within the discipline: business history, cooperative organizations, business development and entrepreneurship, and finally studies of industries with a special focus on the electricity industry. He is also the dynamic chairman of the nonprofit organization Stiftelsen Holistisk Medisin Scandinavia, which aims to support the scientific development, research, and documentation of complementary and holistic medicine in Scandinavia. E-mail: niels.j.andersen@bi.no. Website: www.bi.no/users/fgl93013/

**Joav Merrick, MD, DMSc,** is Professor of Child Health and Human Development affiliated with the Zusman Child Development Center and Division of Community Health at the Ben Gurion University, Beer-Sheva, Israel and presently the Medical Director of the Division for Mental Retardation, Ministry of Social Affairs, Jerusalem and the Director of the National Institute of Child Health and Human Development. He has numerous publications in the field of child and human development, rehabilitation, intellectual disability, disability, health, welfare, abuse, advocacy and prevention. Dr. Merrick received the Peter Sabroe Child Award for outstanding work on behalf of Danish Children in 1985 and the International LEGO-Prize ("The Children's Nobel Prize") for an extraordinary contribution towards improvement in child welfare and well being in 1987. E-mail: jmerrick@internet-zahav.net. Website: www.nichd-israel.com
